# Interventions for Improving Sleep in Dementia Informal Caregivers: A Systematic Review and Meta-Analysis

**DOI:** 10.1093/geroni/igaf122.3798

**Published:** 2025-12-31

**Authors:** Jia Yin Ruan, Xiang Qi, Jin Su, Weiyu Mao, Eunjung Ko, Bei Wu

**Affiliations:** New York University, New York, New York, United States; New York University, New York, New York, United States; New York University, New York, New York, United States; University of Nevada, Reno, Reno, Nevada, United States; New York University, New York, New York, United States; NYU Shanghai, Shanghai, China (People’s Republic)

## Abstract

Although interventions are available to improve sleep for informal caregivers of people with dementia, summarized evidence is still lacking. This study aimed to systematically review and quantify the effects of non-pharmacological interventions on sleep in this population. A literature search was conducted on eleven databases from inception up to 30 June 2025 to retrieve randomized controlled trials. Meta-analyses were performed to estimate the mean difference (MD) of Pittsburgh Sleep Quality Index (PSQI) (primary outcome) using a random-effects model. Risk of Bias 2 tool was applied to measure methodological quality. Twenty-one RCTs encompassing 1964 adults (74.94 % female) were included. The interventions examined in this study refer to behavioural intervention, psychoeducational intervention and physical intervention. The pooled results showed that non-pharmacological interventions were superior to passive controls (e.g., waitlist, usual care), for improving sleep quality (MD = 1.265, 95% confidence interval [0.194, 2.355], p = 0.021) regarding the PSQI score. All included trials had a high overall risk of bias. The overall evidence evaluated by the Grading of Recommendations, Assessment, Development, and Evaluations framework was very low. Future research is encouraged to generate robust evidence by developing well-designed non-pharmacological sleep interventions that incorporate the 4 P Model of insomnia (Predisposing, Precipitating, Perpetuating, Protective), process evaluation and treatment fidelity.

